# Objective quantification of suckling biomechanics in infants with and without tongue-tie using hydraulic differential-pressure recording and machine learning

**DOI:** 10.3389/fdgth.2026.1901883

**Published:** 2026-09-03

**Authors:** Lene Dahl Siggaard, Serguei Chiriaev, Anders Kramer Knudsen, Torben Lildholdt

**Affiliations:** 1 Department of Otorhinolaryngology, Head & Neck Surgery, and Audiology, Aalborg University Hospital, Aalborg University, Aalborg, Denmark; 2 Department of Clinical Medicine, Aalborg University, Aalborg, Denmark; 3 Faculty of Engineering, Mads Clausen Institute, NanoSYD, University of Southern Denmark, Sønderborg, Denmark; 4 Cognitech A/S, Herning, Denmark; 5 ENT Research Clinic, Horsens, Denmark

**Keywords:** ankyloglossia, digital health, frenotomy, infant feeding, intraoral pressure, machine learning, simulated nutritive suckling, suckling

## Abstract

**Introduction:**

Objective assessment of infant suckling biomechanics remains limited despite decades of pressure-based measurement research, and tongue-tie (ankyloglossia) is still largely evaluated using semi-quantitative anatomical scoring. We developed a hydraulic differential-pressure device — the electronic baby bottle (EBB) — designed to record real-time net intraoral mechanical load during nutritive suckling through a fluid-filled sensing cavity with a controlled air inclusion coupled to a differential pressure transducer. This study aimed to: (1) describe the EBB measurement principle, (2) develop automated machine learning–based classification of effective suckling activity, and (3) evaluate whether quantitative biomechanical parameters derived from automated analysis demonstrate responsiveness one week after frenotomy.

**Methods:**

Time-series recordings from iterative prototype testing were segmented into overlapping windows and manually annotated to train a supervised classifier using the ROCKET transform implemented in Python (sktime) with ridge classification and cross-validation. Performance was evaluated on an internal held-out test set and an independent external validation set of full-length recordings excluded from model development. Quantitative parameters were computed from automatically identified effective suckling bursts in 25 infants with restrictive tongue-tie recorded before and one week after frenotomy and compared with 10 control infants with normal tongue mobility.

**Results:**

Automated classification achieved >99% accuracy on the internal test set and 92% segment-level classification accuracy on external validation recordings. Mean negative intraoral pressure during effective suckling bursts increased in 19/25 (76%) treated infants one week after frenotomy (paired t-test, *p* = 0.010), shifting toward control values.

**Discussion:**

Hydraulic differential-pressure recording combined with time-series machine learning enables objective and reproducible quantification of nutritive suckling activity and detects short-term functional change following frenotomy in most infants with restrictive tongue-tie. This framework supports future work on normative reference ranges, standardized digital metrics, and data-driven diagnostic thresholds for infant feeding dysfunction.

## Introduction

Breastfeeding is widely recognized as the biologically optimal method of infant feeding and is associated with substantial short- and long-term health benefits for both infant and mother. Protective effects against gastrointestinal and respiratory infections, improved neurodevelopmental outcomes, and reduced risk of chronic disease later in life have been consistently documented in large systematic reviews and meta-analyses (1, 2). Consequently, exclusive breastfeeding for the first six months of life is recommended by the World Health Organization and numerous national health authorities (1).

Despite these recommendations, breastfeeding difficulties remain common during the early postnatal period. Cohort studies indicate that a substantial proportion of mothers experience significant challenges related to latch, nipple pain, ineffective milk transfer, and prolonged feeding sessions, all of which may contribute to early cessation of exclusive breastfeeding (3–5). Among the anatomical factors implicated in breastfeeding dysfunction, ankyloglossia (tongue-tie) has attracted increasing attention. Ankyloglossia is characterized by a restrictive lingual frenulum that may limit tongue elevation and anterior excursion. Prevalence estimates vary between 4% and 11% of newborns, depending on diagnostic criteria (6, 7). While not all cases are clinically significant, restricted tongue mobility has been associated with impaired latch, maternal nipple trauma, reduced milk transfer, and compensatory feeding behaviors (8–10).

Frenotomy, a minor surgical release of the lingual frenulum, has been shown to provide short-term improvement in breastfeeding symptoms in selected infants (8, 11). However, controversy persists regarding indications, diagnostic thresholds, and long-term outcomes (6, 8). A central challenge in this debate is the absence of objective, reproducible criteria for assessing functional impairment. Current diagnostic tools primarily rely on visual inspection of tongue mobility and structured scoring systems such as the Hazelbaker Assessment Tool for Lingual Frenulum Function or the Bristol Tongue Assessment Tool (12, 13). Although useful, these tools are inherently semi-quantitative and susceptible to inter-observer variability. Moreover, they focus predominantly on anatomical appearance and observable movement rather than on the dynamic biomechanics of nutritive suckling.

Nutritive suckling is a highly coordinated sensorimotor process involving rhythmic generation of negative intraoral pressure combined with compressive forces on the nipple, synchronized with swallowing and respiration (14, 15). Direct measurement of intraoral vacuum during breastfeeding has demonstrated that effective milk transfer depends on adequate generation of sub-atmospheric pressure (14). Over the past decades, several technical approaches have been developed to quantify aspects of suckling dynamics. These include direct intraoral pressure measurement systems (14, 16), pacifier-based pneumatic pressure converters (17–19), displacement sensors embedded in artificial nipples (20), and more recently, sensorized pacifiers combined with computational signal analysis and machine learning (21).

Although these systems have contributed substantially to research on infant feeding physiology, translation into routine clinical diagnostics has been limited. Pneumatic pacifier-based systems rely on air-filled cavities to convert mechanical load into measurable pressure changes. Due to the high compressibility of air, such systems exhibit reduced sensitivity, non-linear response characteristics, and delayed temporal fidelity, potentially compromising accurate representation of rapid pressure fluctuations during suckling (17–19). In addition, many existing devices quantify only a single dynamic component — typically intraoral vacuum — without capturing the net mechanical interaction between suction and compressive forces. Finally, lack of standardized signal analysis pipelines has impeded establishment of clinically meaningful reference values.

To address these limitations, we developed a hydraulic differential-pressure-based measurement system, referred to as the electronic baby bottle (EBB). In contrast to pneumatic systems, the EBB utilizes a fluid-filled sensing cavity integrated within a commercial bottle nipple. Owing to the substantially higher bulk modulus of water compared with air, hydraulic coupling enables more sensitive and linear conversion of biomechanical load into measurable pressure changes. The system records relative pressure variations that reflect the net mechanical load generated by the combined action of intraoral vacuum and tissue compression during simulated nutritive suckling.

In a prior multicenter clinical investigation (Study A), the EBB was introduced into routine ENT practice to evaluate perceived clinical utility and associations with caregiver-reported outcomes. Although ENT specialists rated the device as clinically useful, interpretation of the graphical pressure recordings proved variable across observers and clinical settings, underscoring the need for objective and automated signal analysis (22).

The present study (Study B) therefore had three primary aims. First, to provide a detailed technical description of the hydraulic differential-pressure measurement principle underlying the EBB. Second, to develop and validate a computerized signal analysis framework based on supervised machine learning to enable objective identification of effective suckling activity. Third, to evaluate whether quantitative biomechanical parameters derived from automated analysis demonstrate measurable responsiveness following frenotomy in infants with restrictive tongue-tie.

By integrating sensor engineering, time-series analysis and clinical validation within a unified framework, this study seeks to contribute to the emerging field of digital health technologies for objective assessment of infant feeding dysfunction.

## Materials and methods

### Study population and clinical protocol

This technical validation study (Study B) was conducted following the multicenter clinical utility investigation (Study A) (22). Recordings were obtained from a prospectively enrolled cohort of 35 infants younger than three months of age referred to three participating ENT clinics for evaluation of suspected tongue-tie. Twenty-five infants were clinically diagnosed with restrictive ankyloglossia and underwent frenotomy according to standard clinical indications. Tongue-tie severity was additionally graded before and after frenotomy using the Tongue-tie and Breastfed Babies Assessment Tool (TABBY) scoring system. The infants were recorded with the EBB immediately prior to the procedure and again one week postoperatively. Ten infants with normal tongue mobility, assessed using established clinical scoring systems (12, 13), served as controls and were recorded once.

Infants with craniofacial anomalies, neurological disorders, prematurity below 34 weeks’ gestation, or other conditions affecting feeding biomechanics were excluded. All caregivers provided written informed consent.

The study was approved by the Central Denmark Region Committees on Health Research Ethics (approval no. 1-10-72-155-22) and conducted in accordance with the Declaration of Helsinki.

### Measurement principle of the electronic baby bottle (EBB)

#### Conceptual background and limitations of pneumatic systems

Objective quantification of infant suckling has historically relied on pressure-based systems, most commonly involving pacifiers connected to pressure transducers through air-filled cavities and tubing (17–19). In such configurations, mechanical forces generated by intraoral suction and tissue compression deform the pacifier wall, resulting in pressure changes within the enclosed air cavity. These pressure fluctuations are transmitted to a sensor and converted into electrical signals.

While these systems have provided valuable physiological insights (14, 16), their clinical translation has been limited. A fundamental limitation lies in the compressibility of air. The high compressibility results in reduced sensitivity to small external loads, non-linear pressure-volume relationships, and temporal damping of rapid pressure oscillations (17–19). Moreover, elastic deformation of the pacifier walls may introduce additional mechanical artefacts, compromising accurate representation of the net biomechanical interaction between suction and compression.

In addition, most existing devices quantify either intraoral vacuum or mechanical compression separately, rather than the combined net mechanical load acting on the nipple during suckling (14, 20).

### Hydraulic differential pressure approach

To overcome these limitations, the EBB employs hydraulic coupling for force transmission. The sensing principle is based on a fluid-filled cavity integrated within the nipple of a commercially available feeding bottle (Figure 1a). The conceptual design and technical solutions underlying this approach are disclosed in European patent EP4436476.

**Figure 1:**
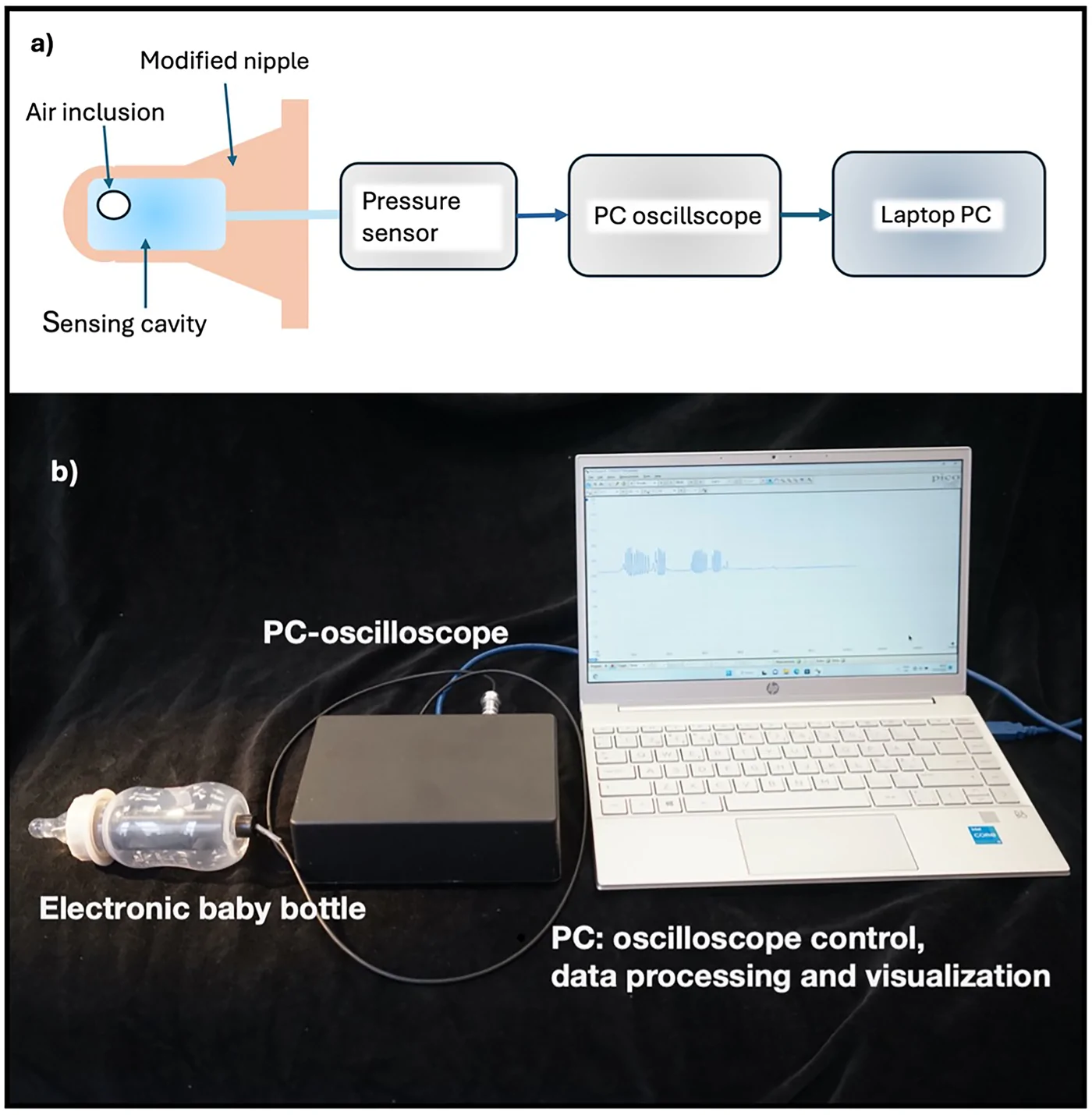
Schematic diagram **(a)** and actual measurement setup **(b)** of the system used in this study.

In the configuration used in the present study, a sensing cavity with a total volume of 1 mL was fabricated within the silicone nipple. The cavity was filled with 0.9 mL of deionized water, leaving a controlled air inclusion of 0.1 mL. The presence of a small air bubble serves to modulate overall compliance and approximate the softness of a maternal breast nipple while maintaining high transduction sensitivity.

The physical rationale for hydraulic coupling is grounded in the difference in bulk modulus between water and air. The bulk modulus of water is approximately 2.2 GPa, which is roughly 20,000 times greater than that of air under standard conditions (23). Consequently, water exhibits minimal volumetric compression under applied load. Compared with pneumatic systems, hydraulic filling ensures substantially greater sensitivity and more linear conversion of external mechanical forces into measurable pressure changes within the cavity.

The cavity was fluidically connected via a short rigid conduit to a Honeywell SSCDRRV015PDAA5 differential pressure sensor configured as a gauge sensor measuring pressure inside the cavity relative to atmospheric pressure. The sensor output was acquired using a Pico Technology PicoScope 2206B interfaced with a laptop computer (Figure 1b). The system was calibrated prior to clinical use.

At any given time point, the measured pressure reflects the net mechanical load on the nipple surface arising from the combined action of negative intraoral pressure (suction) and compressive forces exerted by the tongue and oral tissues. Thus, the signal represents an integrated biomechanical response rather than an isolated measurement of vacuum alone.

### Signal acquisition and preprocessing

Recordings were obtained while infants were held in a natural feeding position. Recordings were performed without milk transfer to standardize mechanical conditions and eliminate flow-dependent confounding; we consider this a controlled functional test rather than a full replication of breastfeeding. Real-time, non-invasive quantification of milk transfer volume associated with individual suckling bursts is not currently achievable with existing technology. This consideration further motivated our decision to standardize testing without milk transfer. When necessary, a drop of 20% sucrose solution was applied to the nipple to facilitate engagement and rhythmic suckling, consistent with established infant feeding research methodologies (17).

Pressure-time signals were continuously recorded and stored for offline analysis. Raw signals were visually inspected to identify recording artefacts. Baseline correction was applied where appropriate to compensate for drift.

Simulated nutritive suckling typically occurred in bursts characterized by rhythmic oscillations dominated by negative pressure peaks (14, 15). To quantify overall biomechanical activity, time-series recordings were segmented into shorter windows to enable both manual annotation and automated classification.

Each data file was labelled, and a time window was slided into overlapping segments of 2,000 datapoints with a stride of 500 datapoints. This overlap ensured that transient transitions between effective and ineffective suckling were not lost and allowed each data point to contribute to up to four segments, improving classifier robustness.

Within identified suckling bursts, mean negative pressure and pressure-time integrals were calculated as candidate quantitative parameters.

### Manual annotation of effective suckling activity

During the iterative testing of various prototypes of the EBB, approximately 250 infants had been recorded one or more times using comparable prototype configurations. These recordings formed the basis for constructing a labelled dataset for supervised machine learning.

To be labelled as effective, at least 1,500 of the 2,000 data points within a segment were required to meet criteria for effective suckling. Segments not meeting this threshold were excluded from the training dataset. In total, 8,864 annotated segments were included.

Annotations were produced by a single trained annotator using a predefined protocol designed to standardize identification of burst onset/offset, peak morphology, and pause duration. Representative annotated recordings are provided as Supplementary Material to illustrate the annotation protocol applied. Formal inter-rater reliability (e.g., Cohen's kappa) was not assessed in this study.

### Machine learning model development

To enable automated and objective classification of suckling activity, a supervised machine learning approach was implemented using the RandOm Convolutional KErnel Transform (ROCKET), a state-of-the-art method for time-series classification (24). ROCKET applies a large number of randomly parameterized convolutional kernels to transform raw time-series data into a high-dimensional feature space, after which a linear classifier can be trained efficiently.

The annotated dataset was divided into a training set comprising 6,648 segments and a test set comprising 2,216 segments. Model training and evaluation were performed using the Python library sktime (25). A ridge regression classifier was trained on the transformed feature space. Ten-fold cross-validation was used during model development to minimize overfitting.

### Model evaluation and external validation

Model performance was first evaluated on the held-out internal test dataset using standard classification metrics, including accuracy, sensitivity and specificity. To assess generalizability, an independent set of 11 full-length time-series recordings, excluded from both training and internal testing, was used for external validation. Automated classification was applied across entire recordings to identify effective suckling activity longitudinally.

Confusion matrices were constructed for both internal testing and external validation datasets. Classification performance on the external dataset was used to estimate real-world applicability in previously unseen recordings.

Given the inherent class imbalance between effective suckling segments (minority class) and ineffective/pause segments (majority class), reflecting the physiological reality that suckling bursts are interspersed with longer inter-burst pauses, model robustness was assessed primarily through 10-fold cross-validation rather than explicit resampling or class weighting. Precision and F1-scores for minority (effective suckling) class are reported alongside accuracy, sensitivity and specificity for both the internal and external datasets (Figures 3, 4) to allow assessment of performance beyond simple accuracy.

### Statistical analysis

Quantitative biomechanical parameters were analyzed using Python statistical libraries. The primary outcome parameter was mean negative intraoral pressure during automatically identified effective suckling bursts.

For infants undergoing frenotomy, pre- and post-intervention values were compared using paired two-sided t-tests after assessment of normality.

Statistical significance was defined as *p* < 0.05. Confidence intervals were calculated at the 98% level, consistent with the two-sided significance threshold of *p* < 0.02 applied to the primary outcome comparison.

## Results

### Signal characteristics and variability

Continuous pressure-time recordings revealed substantial heterogeneity in suckling dynamics across infants. In control infants with normal tongue mobility, simulated nutritive suckling was typically characterized by rhythmic, temporally stable oscillations dominated by reproducible negative pressure peaks separated by short inter-burst pauses. These oscillatory patterns were consistent across bursts within individual recordings.

In contrast, recordings from infants with restrictive tongue-tie frequently exhibited irregular pressure fluctuations, variable burst duration, reduced negative peak amplitude, and intermittent pauses. In several cases, suction cycles appeared fragmented, with inconsistent peak morphology and variable inter-cycle intervals. Motion artefacts and brief periods of non-suckling activity were occasionally observed in both groups but were more prevalent in infants with feeding difficulties.

Visual inspection alone proved insufficient for consistent classification of effective versus ineffective suckling activity. Inter-recording variability and transitional patterns within bursts made manual identification of boundaries between effective and ineffective segments subjective and prone to inconsistency. These observations supported the need for automated signal classification.

Representative examples of machine learning–based automated annotation applied to full-length recordings are shown in Figure 2.

**Figure 2:**
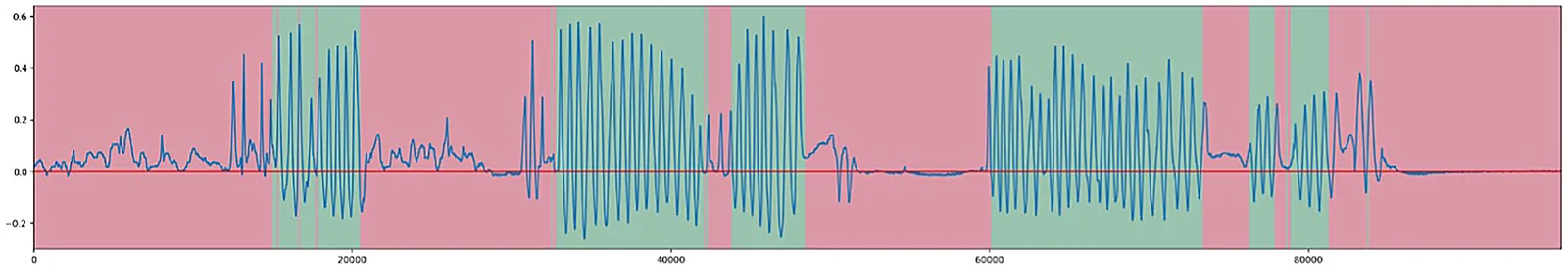
Example of machine learning-based annotation. The *X*-axis represents time, and the *Y*-axis represents sensor output. Green background indicates periods classified as effective suckling, while red background denotes pauses or ineffective activity.

### Performance of automated classification

The supervised machine learning model based on the ROCKET transform demonstrated high discriminatory performance. On the internal test dataset (*n* = 2,216 segments), overall classification accuracy exceeded 99.6%. Sensitivity was 0.986 and specificity 0.999, indicating robust identification of both effective and ineffective suckling segments, with only eight misclassifications (two false positives, six false negatives). The confusion matrix for the internal test set is shown in Figure 3.

**Figure 3:**
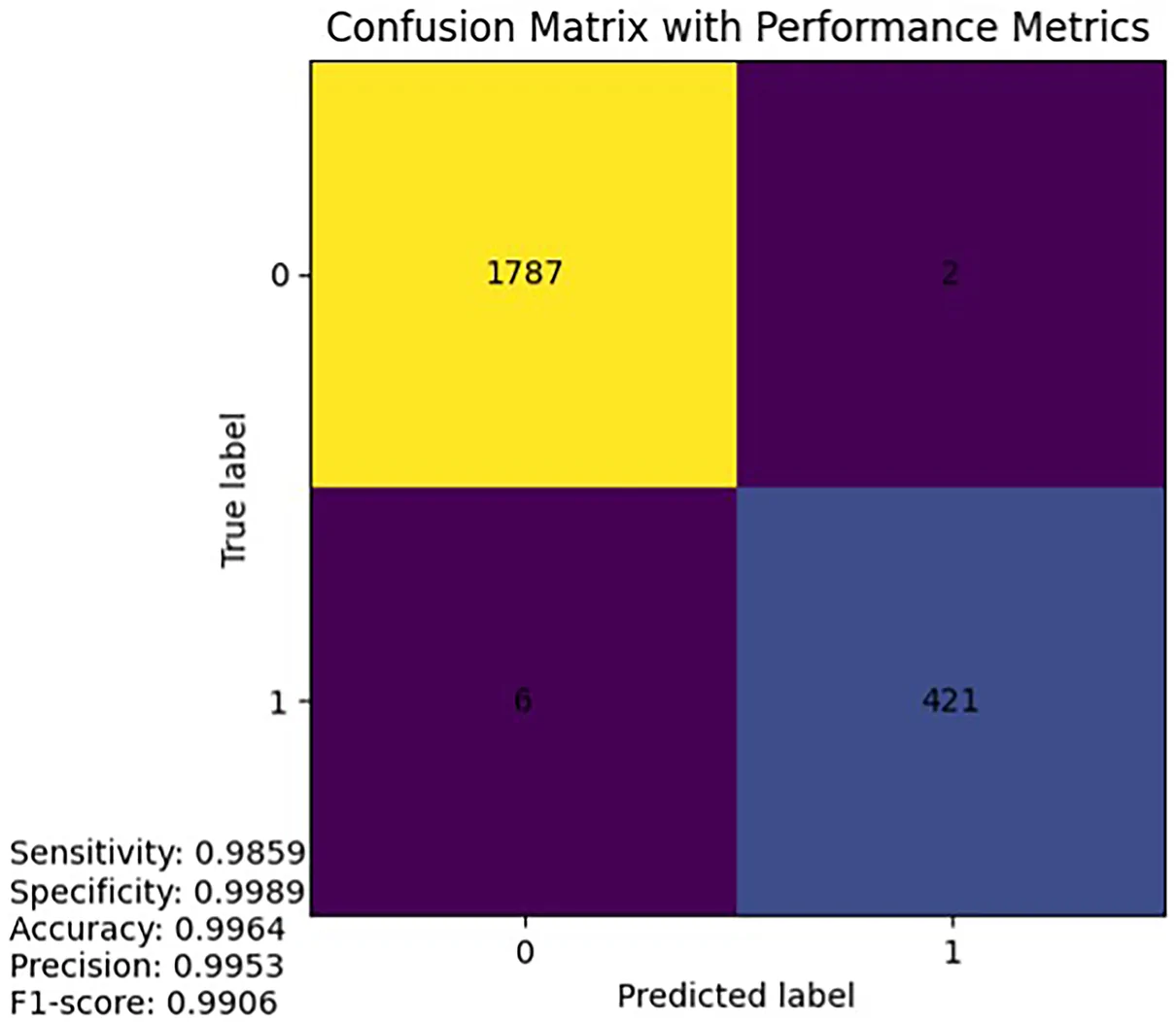
Confusion matrix for the ROCKET classifier evaluated on the internal held-out test set (*n* = 2,216 segments), showing absolute counts. Label 0 = ineffective suckling; Label 1 = effective suckling. The model correctly classified 1,787 ineffective and 421 effective segments, with only 8 misclassifications (2 false positives, 6 false negatives). Overall classification accuracy was 99.6% (sensitivity 0.986, specificity 0.999, precision 0.995, F1-score 0.991).

To assess generalizability, an independent set of 11 full-length recordings, excluded from both training and internal testing, was used for external validation. The model achieved an overall accuracy of 91.8% for segment-level classification across the annotated evaluation set (sensitivity 0.914, specificity 0.921, F1-score 0.904). Misclassifications were primarily observed in transitional segments between effective bursts and pauses, where signal morphology changed gradually rather than abruptly. The confusion matrix for the external validation dataset is shown in Figure 4.

**Figure 4:**
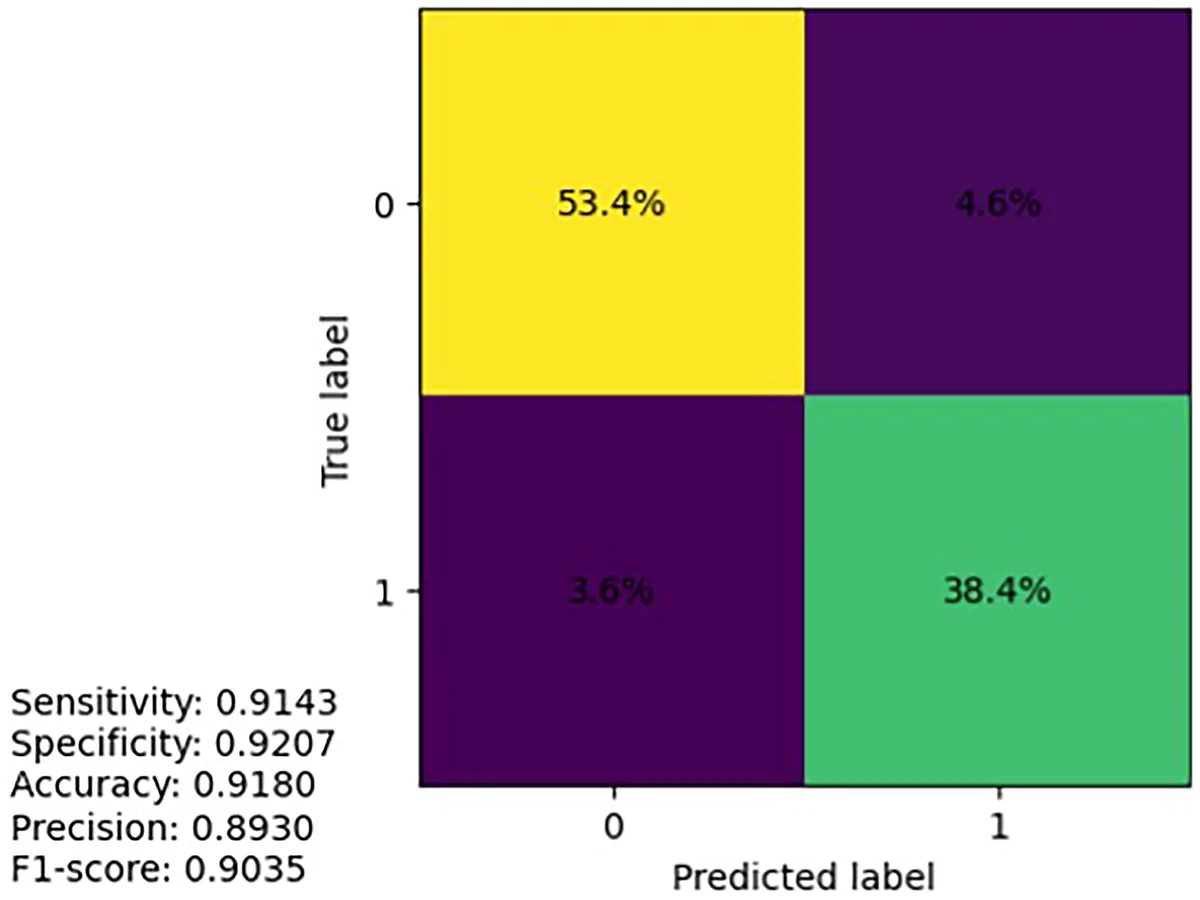
Confusion matrix for the ROCKET classifier evaluated on the independent external validation set (11 full-length recordings), showing the proportion of all classified time points. Label 0 = ineffective suckling; Label 1 = effective suckling. The model correctly classified 53.4% of time points as ineffective and 38.4% as effective, with 3.6% false negatives and 4.6% false positives. Overall classification accuracy was 91.8% (sensitivity 0.914, specificity 0.921, precision 0.893, F1-score 0.904).

A per-recording breakdown of external validation performance (Supplementary Table S1) showed accuracy ranging from 87.7% to 95.9% across the 11 recordings, with no single recording showing markedly poorer performance than the others. This indicates that misclassification was distributed across recordings rather than concentrated in a small subset of infants.

Although a moderate degree of overfitting was observed, as expected when optimizing model parameters on training data, the reduction from internal to external accuracy (99.6%–91.8%) indicates that the model retains strong generalization capability under realistic clinical recording conditions. This reduction is attributable in part to a methodological difference between the internal and external datasets: internal test segments were selected using a ≥75% single-class purity criterion, whereas external validation was performed on continuous, unsegmented full-length recordings that necessarily include transitional intervals between effective and ineffective suckling. As these transitional segments were, by design, underrepresented in the training data, they disproportionately contributed to external misclassification.

### Quantitative biomechanical parameters

Following automated classification of effective suckling segments, quantitative biomechanical parameters were calculated within identified bursts. The primary parameter analysed was mean negative intraoral pressure during effective suckling activity. Pressure values are reported as sensor output units (relative gauge signal) because the system was optimized for within-device comparability. Absolute intraoral vacuum calibration will be addressed in future directions.

### Pre- and post-frenotomy comparison

Among the 25 infants undergoing frenotomy, 19 (76%) demonstrated an increase in mean negative intraoral pressure at one-week follow-up compared with baseline. Five infants showed minimal change or slight reduction. The group mean value prior to frenotomy was −0.08 (sensor output units). One week postoperatively, the mean value increased to −0.10. A paired two-tailed t-test demonstrated a significant change in intraoral pressure one week after frenotomy (mean difference = −0.022, SD = 0.048, t(24) = −2.79, *p* = 0.010). The magnitude of change varied across individuals. In several infants with markedly reduced baseline suction amplitude, postoperative recordings demonstrated both increased peak depth and improved rhythmic stability. In others, improvement was primarily reflected in increased burst duration rather than peak amplitude.

### Comparison with control group

Control infants exhibited a mean negative intraoral pressure of −0.14 (sensor output units). While postoperative values in the tongue-tie group shifted toward control levels, complete normalization was not observed in all cases. The difference between preoperative tongue-tie recordings and controls was statistically significant. Postoperative values remained lower than controls but demonstrated a clear directional shift.

Group-level data are summarized in Table 1.

**Table 1:** Mean negative intraoral pressure during effective suckling bursts before and after frenotomy in infants with restrictive tongue-tie.

Patient	Before frenotomy (sensor output units)	After frenotomy (sensor output units)	Change	Direction
#01	−0.049	−0.087	−0.038	↑
#02	−0.071	−0.123	−0.052	↑
#03	−0.076	−0.083	−0.007	↑
#04	−0.144	−0.159	−0.015	↑
#05	−0.115	−0.127	−0.011	↑
#06	−0.082	−0.065	+0.016	↓
#07	−0.044	−0.021	+0.024	↓
#08	−0.019	−0.085	−0.066	↑
#09	−0.040	−0.052	−0.013	↑
#10	−0.136	−0.146	−0.010	↑
#11	−0.038	−0.175	−0.137	↑
#12	−0.172	−0.203	−0.031	↑
#13	−0.051	−0.099	−0.048	↑
#14	−0.089	−0.172	−0.082	↑
#15	−0.031	−0.022	+0.009	↓
#16	−0.159	−0.171	−0.012	↑
#17	−0.162	−0.134	+0.029	↓
#18	−0.094	−0.062	+0.033	↓
#19	−0.032	−0.101	−0.069	↑
#20	−0.020	−0.035	−0.015	↑
#21	−0.047	−0.078	−0.031	↑
#22	−0.067	−0.085	−0.018	↑
#23	−0.022	−0.067	−0.046	↑
#24	−0.079	−0.087	−0.008	↑
#25	−0.083	−0.042	+0.041	↓
Summary statistics				
Group mean	−0.075	−0.098	−0.022	
SD	0.048	0.054	0.048	
98% CI of change			[−0.042; −0.002]	

Paired t-test: t(24) = −2.79, *p* = 0.010; 19/25 (76%) showed improvement.

Control group mean (*n* = 10): −0.140 (single recording).

All values are mean negative intraoral pressure during automatically identified effective suckling bursts, expressed as sensor output units (relative gauge signal). More negative values indicate stronger suction. ↑ = increased negative pressure (improvement); ↓ = reduced or unchanged negative pressure.

### Representative signal patterns

Representative pressure-time traces illustrate distinct biomechanical phenotypes. In control infants, effective suckling bursts displayed stable periodic oscillations with consistent peak morphology and relatively uniform amplitude. In infants with restrictive tongue-tie prior to frenotomy, several characteristic patterns were observed: reduced peak amplitude, irregular cycle timing, shortened burst duration, and intermittent pauses within bursts.

Post-frenotomy recordings in responsive infants showed increased negative peak depth and improved rhythmicity. In some cases, the postoperative pattern closely approximated control morphology, whereas in others improvement was partial.

Examples of representative recordings are shown in Figures 5, 6. Figure 5 depicts a graph demonstrating a consistent suckling pattern characterized by oscillatory bursts of positive and negative pressure in control infants.

**Figure 5:**
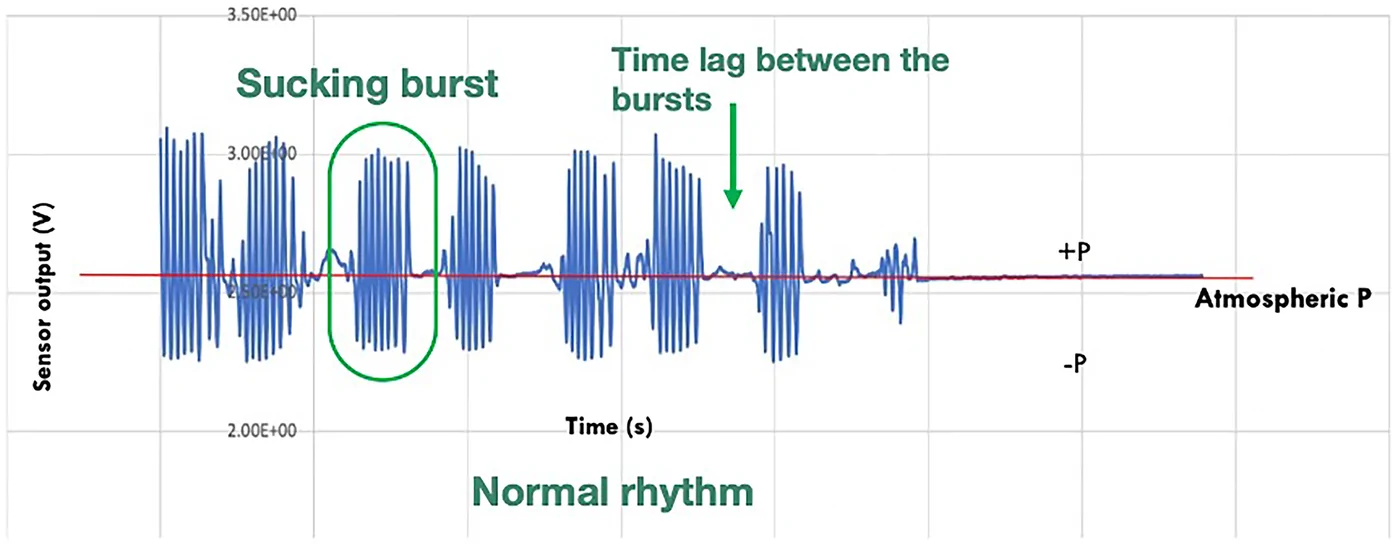
Consistent suckling pattern in a healthy infant with no feeding issues. The positive pressure (“+P”) peaks reflect the compression of the nipple, while the negative pressure (“-P”) peaks represent suction inside the oral cavity. The regular oscillations indicate efficient feeding biomechanics, where the infant alternates between compression and suction to extract milk effectively.

**Figure 6:**
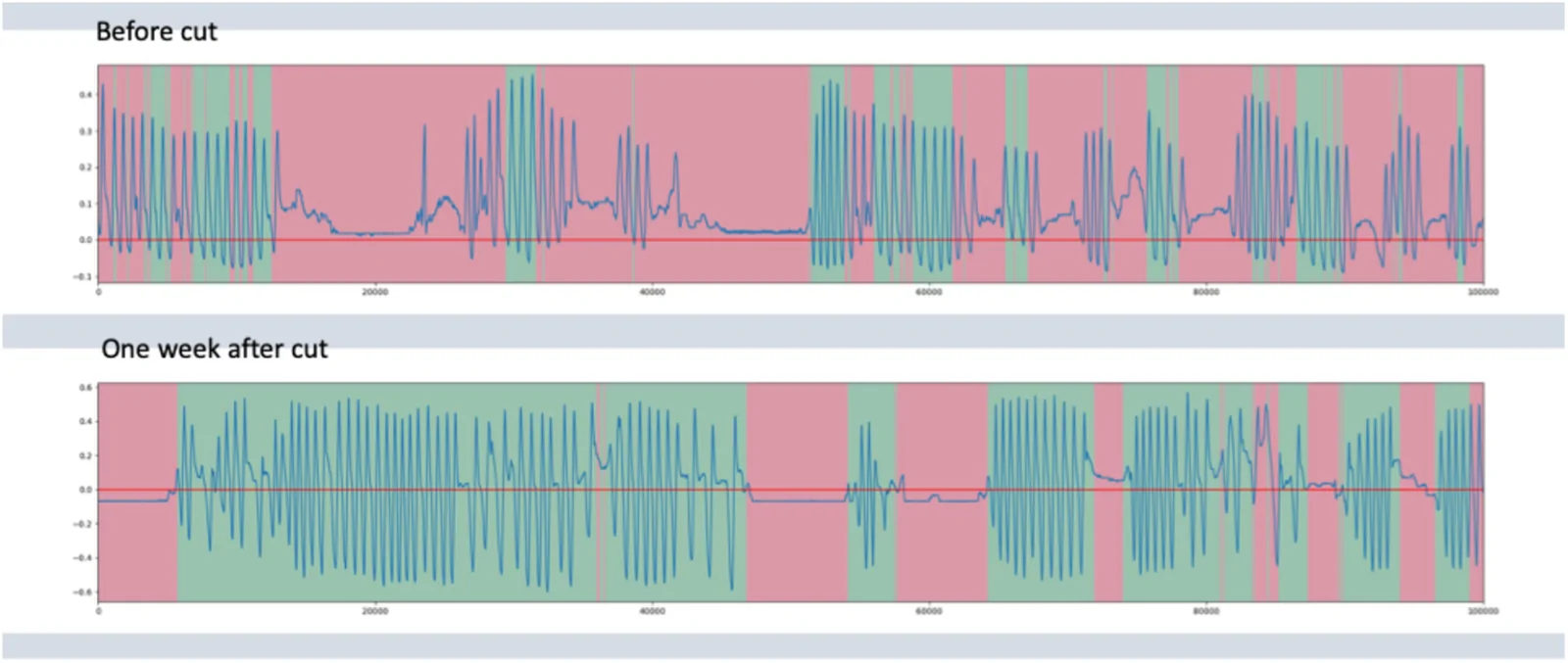
Machine learning-annotated recordings from tongue-tie infant before and after frenotomy. The suckling bursts have shifted predominantly into the negative pressure region following a frenotomy procedure. Positive pressure peaks in graph b are less pronounced compared to graph a.

The example in Figure 6 depicts two graphs (a and b) from a tongue-tie infant pre- and postoperatively, respectively.

In summary, automated signal classification enabled objective identification of effective suckling activity with high internal and external validity. Quantitative analysis demonstrated statistically significant increases in mean negative intraoral pressure following frenotomy in most infants with restrictive tongue-tie. Although postoperative values did not universally reach control levels, a consistent directional shift toward stronger suction generation was observed.

## Discussion

This study demonstrates that hydraulic differential pressure recording combined with automated machine learning–based signal analysis enables objective and reproducible quantification of simulated nutritive suckling biomechanics in infants. The principal findings were threefold. First, visual interpretation of pressure-time recordings proved insufficiently reliable for consistent classification of effective suckling activity. Second, supervised time-series classification using the ROCKET framework achieved high internal accuracy and robust external validation performance. Third, quantitative analysis of mean negative intraoral pressure revealed a statistically significant increase following frenotomy in most infants with restrictive tongue-tie, with postoperative values shifting toward those observed in controls.

Together, these findings support the feasibility of translating pressure-based suckling assessment from a primarily research-oriented tool into a structured digital diagnostic framework.

### Technological implications

A central innovation of the present system lies in the use of hydraulic rather than pneumatic coupling for force transduction. Previous pacifier-based systems have relied on air-filled cavities to convert mechanical load into pressure changes (18–20). While these designs have advanced understanding of infant feeding physiology, the high compressibility of air inherently reduces sensitivity and may dampen rapid pressure oscillations. In contrast, the substantially higher bulk modulus of water allows more faithful conversion of mechanical deformation into measurable pressure change (23).

By integrating a fluid-filled cavity with a controlled air inclusion, the EBB combines high transduction sensitivity with adjustable compliance. This design aims to approximate physiological nipple softness while preserving linearity and temporal resolution. Importantly, the measured signal reflects the net mechanical load generated by the interaction between intraoral vacuum and compressive forces. In this respect, the system captures a biomechanical composite rather than isolating vacuum generation alone.

From a digital health perspective, the distinction is clinically relevant. A measurement principle that reliably reflects the integrated biomechanics of simulated nutritive suckling provides a more comprehensive basis for downstream computational analysis and potential threshold development.

### Need for automated signal interpretation

In Study A, clinicians rated the graphical output as clinically informative; however, inter-observer variability and heterogeneity of signal morphology limited reproducibility (22). The present findings confirm that manual interpretation alone is insufficient for standardized classification. Transitional segments between bursts, interspersed pauses, and variable amplitude patterns introduce ambiguity that cannot be resolved reliably by visual inspection alone.

The implementation of a supervised time-series classifier addresses this limitation. ROCKET has emerged as a highly efficient and accurate method for time-series classification across domains (25). Its ability to transform raw signals into a discriminative feature space without extensive handcrafted feature engineering makes it particularly suitable for physiological waveform analysis given the limited number of annotated time series. The high classification accuracy observed in internal testing and the robust performance in external validation suggest that effective suckling activity can be identified objectively and consistently.

Importantly, misclassifications in the external dataset were predominantly observed in transitional regions rather than during stable bursts. This pattern indicates that the model captures core burst morphology reliably but may be sensitive to gradual changes in signal dynamics. Such behavior is consistent with the biological continuum between effective and ineffective activity and may reflect physiological variability rather than algorithmic failure. Accordingly, a larger number of children tested may increase the accuracy. Consistent with this, per-recording analysis of the external validation set (Supplementary Table S1) confirmed that classification accuracy remained within a relatively narrow range (87.7–95.9%) across infants, supporting the interpretation that residual error reflects the inherent ambiguity of transitional signal segments rather than infant-specific model failure.

### Biomechanical responsiveness following frenotomy

The significant increase in mean negative intraoral pressure following frenotomy in 76% of treated infants provides objective evidence of altered suckling biomechanics. Previous studies have reported clinical improvement after frenotomy based primarily on maternal pain reduction, latch scores, and feeding duration (8, 11). However, objective quantification of suction dynamics has been limited.

Generation of negative intraoral pressure is a critical determinant of milk extraction during breastfeeding (14, 15). Restricted tongue mobility may reduce the infant's ability to generate and sustain sub-atmospheric pressure, thereby compromising milk transfer efficiency. The observed postoperative increase in negative pressure amplitude is physiologically plausible and consistent with improved tongue elevation and seal formation against the palate.

Notably, postoperative values did not uniformly reach control levels. This finding aligns with the heterogeneity of ankyloglossia severity and suggests that anatomical release alone may not fully normalize complex neuromotor feeding patterns in all infants. Additionally, adaptive motor learning following frenotomy may require more than one week to stabilize. This pattern is consistent with our companion clinical investigation, in which feeding-symptom scores improved after frenotomy and approached, without fully matching, control-group levels (22).

The presence of five infants without measurable improvement underscores that frenotomy does not uniformly alter biomechanical output. Objective quantification may therefore assist in refining selection criteria and in distinguishing anatomical restriction from multifactorial feeding dysfunction.

### Integration within digital health frameworks

The combination of sensor engineering, structured signal segmentation and supervised machine learning represents a digital health approach to infant feeding assessment. Rather than relying solely on anatomical scoring systems, this framework introduces quantitative biomechanical parameters that can be standardized, stored and analyzed longitudinally.

Establishing normative reference ranges for negative intraoral pressure and burst characteristics could enable development of clinically actionable thresholds. In addition, real-time classification algorithms may ultimately support point-of-care decision-making. The modular nature of the system permits integration with future predictive models or multimodal datasets incorporating weight gain trajectories or feeding duration metrics. Such developments align with broader trends toward objective, data-driven diagnostic augmentation in pediatric medicine.

### Strengths and limitations

A major strength of this study is the integration of a physically grounded measurement principle with supervised machine learning and clinical responsiveness testing. The use of an external validation dataset strengthens confidence in model generalizability.

Several limitations should be acknowledged. First, annotations were generated by a single trained annotator following a predefined protocol: formal inter-rater reliability (e.g., Cohen's kappa) was not assessed, and the resulting “ground truth” therefore reflects one observer's interpretation rather than a validated consensus standard. Given that supervised classifier performance is fundamentally bounded by label quality, this is considered a significant limitation of the present study, and multi-annotator validation is planned as a priority for future work. Second, we prioritized characterization of misclassification along the time axis (i.e., identifying whether errors cluster in transitional segments) over presentation of precision-recall curves, as the temporal location of errors was considered more clinically informative than aggregate threshold-independent performance metrics for this application. Future work with larger and more balanced datasets should include formal precision-recall analysis and explicit class-weighting strategies. Third, recordings were performed without active milk transfer to standardize conditions. This may not fully replicate breastfeeding biomechanics. Fourth, the control group, although confirmed to have normal tongue mobility using validated scoring tools, was recruited from infants referred for suspected tongue-tie rather than from an unselected, healthy community cohort. This may introduce selection bias, and standardized feeding-symptom data were not systematically collected for comparison between groups. A community-based asymptomatic control cohort would strengthen future validation of normative reference values. We note that recruitment of such a control cohort has also proven challenging in our related clinical work (22), underscoring that this is a recurring methodological challenge rather than an oversight specific to the present study. Fifth, sample size was modest, and the follow-up interval was limited to one week. Sixth, although tongue-tie severity was assessed using the TABBY scoring system, formal correlation analysis between severity grading and quantitative pressure metrics was not performed in the present study; furthermore, TABBY scoring showed limited consistency across our cohorts, warranting cautious interpretation of this metric and further investigation in adequately powered future studies. Finally, pressure values were expressed in relative units; translation to absolute physiological pressure values would require further calibration against established intraoral vacuum measurements (14).

### Future directions

Future research should focus on expanding normative datasets across age groups, evaluating longitudinal changes in suckling biomechanics, and integrating objective pressure metrics with clinical outcome measures such as weight gain and breastfeeding duration. Assessment of inter-rater annotation reliability and exploration of unsupervised or semi-supervised learning approaches may further refine classification robustness. In addition, comparative studies against established direct intraoral vacuum measurement systems would help situate hydraulic differential pressure recording within the broader physiological literature.

## Conclusions

Hydraulic differential pressure recording combined with automated time-series classification enables objective assessment of simulated nutritive suckling biomechanics in infants. The system demonstrates measurable biomechanical responsiveness following frenotomy and provides a reproducible digital framework for quantifying feeding dynamics. With further validation and normative reference development, such technology may contribute to more consistent evaluation of infants with suspected tongue-tie and related feeding dysfunction.

## Data Availability

The datasets generated and analysed during the current study are not publicly available due to ethical and data protection restrictions involving recordings from infants. Anonymized derived data and model performance outputs may be made available from the corresponding author upon reasonable request and subject to institutional approval. The machine learning pipeline was implemented using the publicly available Python library sktime. Requests to access the datasets should be directed to LS, lenedahlsiggaard@gmail.com.
