# Supplementary material for: Objective quantification of suckling biomechanics in infants with and without tongue-tie using hydraulic differential-pressure recording and machine learning
Source: Front Digit Health. 2026 Sep 3;8:1901883. doi: 10.3389/fdgth.2026.1901883 (PMC13647136; doi:10.3389/fdgth.2026.1901883)
Supplement: Supplementary file 1 [file Table1.docx]

**Supplementary Material**

**Supplementary Table S1.** Per-recording classification performance for the 11 full-length recordings comprising the external validation dataset.

| **Case** | **n (datapoints)** | **Accuracy** | **Sensitivity** | **Specificity** |
| --- | --- | --- | --- | --- |
| #01 | 95,884 | 92.1% | 85.6% | 96.9% |
| #02 | 91,284 | 92.1% | 85.3% | 97.0% |
| #03 | 100,004 | 90.3% | 92.0% | 87.5% |
| #04 | 100,006 | 89.6% | 96.5% | 76.5% |
| #05 | 100,004 | 87.7% | 83.4% | 89.5% |
| #06 | 100,004 | 94.0% | 90.2% | 95.3% |
| #07 | 100,004 | 90.5% | 91.4% | 90.4% |
| #08 | 100,006 | 95.9% | 93.7% | 96.9% |
| #09 | 74,584 | 90.5% | 93.4% | 87.7% |
| #10 | 131,072 | 93.9% | 91.1% | 96.1% |
| #11 | 131,051 | 92.5% | 95.2% | 88.8% |

**Legend:** For each recording, the table reports the number of classified datapoints, overall accuracy, sensitivity, and specificity, together with the corresponding confusion matrix (absolute counts of true positive, true negative, false positive, and false negative classifications for effective vs. ineffective sucking). These per-recording metrics underlie the aggregate external validation performance reported in the main text (Results, "Performance of Automated Classification") and demonstrate that classification accuracy was consistently distributed across recordings (range 87.7–95.9%) rather than concentrated in a small subset of infants.

**Supplementary Figures:** Representative annotated pressure-time recordings for each of the 11 external validation cases, illustrating the machine learning–based classification of effective (green background) versus ineffective/pause (red background) sucking activity across the full length of each recording. These figures illustrate the annotation protocol applied by the trained annotator and the corresponding pattern of model misclassification (concentrated in transitional segments between effective bursts and pauses, rather than within stable bursts), as discussed in the Discussion ("Need for Automated Signal Interpretation").

**Case #01**

| Case #01 | | **True label** | |
| --- | --- | --- | --- |
|  |  | 0 | 1 |
| **Predicted label** | 0 | 53077 | 5907 |
|  | 1 | 1698 | 35202 |

**Case #02**

| Case #02 | | **True label** | |
| --- | --- | --- | --- |
|  |  | 0 | 1 |
| **Predicted label** | 0 | 51480 | 5604 |
|  | 1 | 1595 | 32605 |

**Case #03**

| Case #03 | | **True label** | |
| --- | --- | --- | --- |
|  |  | 0 | 1 |
| **Predicted label** | 0 | 32994 | 5010 |
|  | 1 | 4715 | 57285 |

**Case #04**

| Case #04 | | **True label** | |
| --- | --- | --- | --- |
|  |  | 0 | 1 |
| **Predicted label** | 0 | 26396 | 2310 |
|  | 1 | 8090 | 63210 |

**Case #05**

| Case #05 | | **True label** | |
| --- | --- | --- | --- |
|  |  | 0 | 1 |
| **Predicted label** | 0 | 63597 | 4807 |
|  | 1 | 7491 | 24109 |

**Case #06**

| Case #06 | | **True label** | |
| --- | --- | --- | --- |
|  |  | 0 | 1 |
| **Predicted label** | 0 | 70902 | 2502 |
|  | 1 | 3495 | 23105 |

**Case #07**

| Case #07 | | **True label** | |
| --- | --- | --- | --- |
|  |  | 0 | 1 |
| **Predicted label** | 0 | 84103 | 601 |
|  | 1 | 8899 | 6401 |

**Case #08**

| Case #08 | | **True label** | |
| --- | --- | --- | --- |
|  |  | 0 | 1 |
| **Predicted label** | 0 | 66198 | 2008 |
|  | 1 | 2096 | 29704 |

**Case #09**

| Case #09 | | **True label** | |
| --- | --- | --- | --- |
|  |  | 0 | 1 |
| **Predicted label** | 0 | 33583 | 2401 |
|  | 1 | 4694 | 33906 |

**Case #10**

| Case #10 | | **True label** | |
| --- | --- | --- | --- |
|  |  | 0 | 1 |
| **Predicted label** | 0 | 69516 | 5256 |
|  | 1 | 2804 | 53496 |

**Case #11**

| Case #11 | | **True label** | |
| --- | --- | --- | --- |
|  |  | 0 | 1 |
| **Predicted label** | 0 | 48146 | 3705 |
|  | 1 | 6093 | 73107 |
